# Association between gender norms and attitudes towards abortion among very young adolescents in Kenya and Nigeria: a quantitative study

**DOI:** 10.1136/bmjph-2024-001456

**Published:** 2025-10-07

**Authors:** Matthew Ayodele Alabi, Bamidele Motunrola Bello, Beatrice Maina

**Affiliations:** 1 Programmes, Population Council Nigeria, Abuja, Nigeria; 2 Academy for Health Development (AHEAD), Ile-Ife, Nigeria; 3 Research, African Population and Health Research Center, Nairobi, Kenya

**Keywords:** Public Health, Public Health Practice, Community Health

## Abstract

**Introduction:**

Unsafe abortion is a major cause of death in sub-Saharan African countries. Adolescents are at increased risk due to their high vulnerability to unprotected sex and unplanned pregnancies. Abortion beliefs and attitudes are considered to be partly rooted in traditional views on gender and religious influences. This study is informed by the limited data on gender norm perception and its association with abortion attitudes among adolescents, despite the increasing prevalence of unsafe abortion reported among this group.

**Materials:**

Data for this study were collected as part of a longitudinal survey on gendered socialisation and sexual and reproductive health (SRH) of in-school adolescents aged 10 – 14 (very young adolescents (VYA)) years in Kenya and Nigeria. The study obtained quantitative data from 1912 VYAs using a structured questionnaire. The results presented in this paper are from the quantitative baseline data collected in Kenya and Nigeria.

**Result:**

The study found significant regional differentials in attitudes towards abortion and gender norm perception of the VYA from the two countries. In Nigeria, VYA were more likely to endorse abortion practices relative to their counterparts from Kenya. Factors associated with endorsement of abortion practice were gender norms about sexual double standards and normative heterosexual relations (NHR) in Nigeria and knowledge of where to get a condom, NHR and normative romantic relationship in Kenya.

**Conclusion:**

Gender norms are learnt early. Intervention efforts seeking to promote positive gender norms and attitudes towards SRH must begin with the VYA, consider contextual variations and address knowledge and access to SRH commodities and services.

**WHAT IS ALREADY KNOWN ON THIS TOPIC**

- Previous studies have explored attitudes towards gender norms and abortion among young people and adults, with little known about very young adolescents aged 10–14 years.

**WHAT THIS STUDY ADDS**

- The study contributed to the limited research on gender norms and attitudes towards abortion among very young adolescents.

**HOW THIS STUDY MIGHT AFFECT RESEARCH, PRACTICE AND POLICY**

- The outcome of this study suggests the need to consider early measure that takes into considerationcontextual variations when developing interventions and policies addressing gender norms and attitudes towards abortion in Africa.

## Background

 About 73 million induced abortions take place globally annually.^1^ Approximately 45% of all induced abortions are unsafe, with developing countries bearing the highest burden of unsafe abortion (95%), more so in Africa and Latin America.^1^ Unsafe abortion, according to the WHO, refers to those performed by individuals either lacking the requisite skills or in an environment that does not follow minimal medical standards or both, in addition to highlighting how social and legal contexts influence abortion safety.^2^ Abortion among adolescent girls constitutes a considerable proportion of the abortion rate globally, with 15% of unsafe abortions occurring among adolescent girls under the age of 20 years.^3^ Nearly three-quarters of abortion-induced hospitalisations reported in developing countries are among adolescent girls under the age of 20 years.^4^ Although abortion is considered illegal in several African countries, including Kenya and Nigeria, except on certain stringent conditions such as life-threatening cases, opposition to abortion is considered to be partly rooted in traditional views on gender and religious influences.^5 6^

In Kenya and Nigeria, the attitude towards abortion is generally conservative. While the constitutions of both countries suggest a possibility of women accessing safe abortion,^7^ there remains a negative sociocultural attitude towards abortion in these countries.^8^ The negative attitude towards abortion no doubt can generate undesirable sexual and reproductive health (SRH) outcomes. For instance, a Kenyan study^8^ found a varied and high prevalence of negative attitudes towards abortion influenced by several socioeconomic and cultural variables, including gender, place of residence, level of education and position in society, among others. Similarly, a study examining attitudes and practices of abortion among female in-school adolescents in Lagos, Southwest, Nigeria found over 80% of them had knowledge of abortion, and nearly all of them (99.2%) disapproved of abortion practice. The study found that while knowledge of abortion legality and the procedure was high among the very young adolescents (age 10 – 14) (VYA), they were less likely to approve of the practice relative to their older counterparts in the age group 15 – 19 years.^9^

Gender norms—socially constructed roles, behaviour, activities and attributes considered appropriate for boys/men or girls/women in a particular society —are usually acquired during the early stages of childhood development (especially during the early adolescent period) through social interactions, within a particular setting. The adolescence period is generally seen as a very critical period in an individual’s life, largely because identities are formed during this stage of life, and several attitudes and behaviours become noticeable.^10^ Scholars have argued that gender norms can be acquired at a much earlier childhood stage of development. For instance, it is believed that by age 6, children already possess the ability to distinguish between what it means to be a boy or a girl, and the attributes of each sex.^11 12^ This is in addition to the ability to express appropriate sex-role preferences for themselves and behave in accordance with sex-role standards.^13^

Previous studies in America, Asia and Africa have reported a significant positive association between gender norm perception and attitude towards abortion.^14 15^ In a South African study, attitude towards abortion was associated with socioeconomic and gender ideological dynamics and social status.^16^ Besides, not until recently, most studies on abortion^17 18^ have particularly focused on young women aged 15–24 years and older adults, while neglecting the VYA. Although the prevalence of sexual activity may not be as high among VYA, early adolescence is a stage where sexual activity is initiated and beliefs and norms about sexual relations and activities are internalised.^19^,^21^ Extant literature suggest gender norms and power imbalance relating to women’s SRH are largely influenced by the absence of agency in making SRH decisions, stigma, lack of financial resources and the need to be secretive often result in harmful abortion practices and also delays in seeking care.^22^

However, to the best of our knowledge, studies linking gender norms with attitudes towards abortion in Africa, especially among VYA, remain very sparse. Given the high rate of risky sexual behaviours among older adolescents and the formative role early adolescence plays in shaping norms and attitudes, understanding perceptions of abortion and how these relate to gender norms and SRH among VYA is essential. p Such insights are critical for informing effective programmatic interventions that address the unique SRH needs of this demographic. To this end, this study examined gender norms, perceptions and attitudes towards abortion among VYA in Kenya and Nigeria.

## Material and methods

### Study design/population

The study utilised baseline data from a longitudinal survey on the gendered socialisation of very young in-school adolescents aged 10–14 years and their SRH, conducted in urban informal settlements in Nigeria (Ile-Ife and Modakeke) and Kenya (Korogocho and Viwandani). The main study adopted a concurrent mixed-method research design, which involves collecting both quantitative and qualitative data. However, the results of this study are limited to findings from the quantitative data. The data collection tools were developed and validated in the context of the Global Early Adolescent Study and consisted of three instruments: a health+instrument, a vignette-based instrument and the gender norms scale. The tool has undergone face and content validity assessment and has been pilot-tested in a previous study - Global Early Adolescent Study (GEAS) in Ile-Ife, Nigeria and Korogocho slums, Kenya.^23^

### Data collection

For this study, we used the health+instrument and the gender norm scale to measure gender norms and SRH outcomes among VYA. The health+instrument obtained sociodemographic information and some key health and behavioural factors, including pubertal maturation, romantic relationships, mental health and future expectations. Measures of abortion and the sociodemographic characteristics of early adolescents were obtained from this instrument. The gender-norm scale assessed the gendered perception of adolescents using a series of questions that have a gender undertone. Information collected using this instrument was used to assess early adolescents’ gender-related beliefs. The data collection approach used was very similar for both countries. In Kenya, the interviews were conducted by young people who had received training on how to conduct research with young adolescents and had undergone a research ethics course. Data were collected in school. Interviews were interviewer-based using a modified computer-assisted personal interviewing (CAPI) technique whereby an interviewer was grouped with about five adolescents at every interview session. Each adolescent was assigned a tablet. An interviewer read each question and response option, and the adolescent respondent entered their particular responses on their tablets. Caution was taken to ensure the privacy and confidentiality of individual adolescent responses by ensuring adequate spacing between respondents. Additionally, to ensure that all participants moved at the same pace and to protect participants who might have had some sensitive experiences, such as sexual activity, all filters were removed from the tool, and response options were replaced with relevant not-applicable response options. Same-sex participants were matched with the same-sex interviewers. Similarly, in Nigeria, data were collected by trained interviewers using CAPI, specifically SurveyCTO on tablets, and uploaded to a central data repository, where all data files were maintained in a secure server. The interviewers were young people, specifically fresh university graduates between the ages of 21–26 years old. The interviewers were exposed to 3 days of intensive training on conducting research with the adolescent population, ethical issues, among others, before data collection. The interviewers were also assigned based on sex (male interviewers interviewed male respondents, while female interviewers interviewed female respondents). The same interviewer-based approach was used in both countries, and all interviews were conducted within the school premises. Identifiers were removed from all analytical datasets and the presentation of results.

### Sampling technique

This study uses baseline data to examine the perceptions of VYA towards abortion. Sampling and data collection were done before the provision of any intervention. The study was conducted in seven randomly selected schools serving primarily urban disadvantaged populations within Ife and Modakeke communities in Ile-Ife, an ancient city in Osun State, southwest Nigeria, and four schools in Korogocho and Viwandani informal settlements in Kenya. In both countries, the primary target was in-school adolescents aged 10–14 years. Within the educational system, the majority of these populations are in grades 5 and 6. Hence, all the adolescents in grades 5 and 6 were recruited after obtaining informed consent from their primary caregiver and the adolescent assent. To identify schools serving primarily urban disadvantaged populations, the schools participating in the study in Kenya were purposely identified based on mapping public day primary schools serving the population living within the Nairobi Urban Demographic Surveillance System (NUHDSS). The NUHDSS is a platform that African Population and Health Research Centre (APHRC) has been running in Korogocho and Viwandani informal settlements, in which studies focused on informal settlements are nested.^24^ The four schools selected in Nairobi, two in Korogocho and two in Viwandani served a majority of the population. In Nigeria, the schools were selected from communities defined as urban poor—the major source of livelihood within the communities is employment in the informal sector. Housing structures in these communities are mostly traditional (mud, uncemented). The communities are densely populated and characterised by poor water and sanitation infrastructure. In total, 1,032 in-school VYAs from seven schools in Nigeria and 880 from four schools in Kenya were recruited and interviewed. The sample size was arrived at based on the total census of eligible study participants within the eligible school grades who completed the assent form and whose parents consented to their children participating in the study across the sampled schools.

### Measurement of variable outcome variable

The outcome variable for this study is the attitude towards abortion, measured using the perception of adolescents towards abortion. Three questions were asked, namely: adolescent girls who get pregnant should have an abortion if they are not married; adolescent girls who get pregnant should have an abortion because they are too young to raise children; adolescent girls who get pregnant should have an abortion to stay in school. All three questions were measured on a 5-point Likert scale, ranging from disagree a lot, coded as 1 and agree a lot, coded as 5. However, for ease of analysis, principal component analysis (PCA) was performed and used to generate a single score for all three items and grouped into high and low. High scores indicate acceptance of abortion (and coded as 1), while low score indicates disapproval of abortion (coded as 0). The two categories of high and low inform the basis for acceptance and rejection, following the same response categories.

### Explanatory variables

The principal explanatory variable in this study is gender norms. The overall gender norm aspect of the multidimensional scale comprises three subscales, namely: sexual double standard (SDS) scale, stereotypical view about toughness versus weakness and normative heterosexual relation (NHR). The SDS scale was further divided into three domains, namely: SDS, normative romantic relation (NRR) and masculine sexual prowess (MSP). Each of the scales was measured on a 5-point Likert scale ranging from disagree a lot (1), disagree a little (2), neither agree nor disagree (3), agree a little (4), to agree a lot (5). The reliability analysis of the scales yielded a Cronbach alpha exceeding the minimum recommended value of 0.70 except for MSP subdomain: SDS scale (α=0.89), stereotypical view about toughness versus weakness (α=0.82); normative heterosexual relation scale (α=0.85); SDS subscale (α=0.86); NRR subscale (α=0.78); MSP (α=0.53) and attitude towards abortion (α=0.89). Since the MSP subscale failed to reach the minimum acceptable threshold, it was excluded from further analysis. Other explanatory variables used include sociodemographic characteristics, namely: age, religion (Christian vs Islam) and sex (male vs female), knowledge of where to get condoms and pregnancy prevention methods, with yes or no as the options.

### Ethical consideration

Approval was obtained from all local ethics committees in both countries. In Nigeria, the Principal Investigator’s institution is Academy for Health Development (AHEAD, Ile-Ife, Nigeria). The research protocol was reviewed and approved by the local Health Planning, Research, and Statistics Department, Osun State Ministry of Health, approval no: OSHREC/PRS/569T/149. Approval from the National Health Research and Ethics Committee was not required for the case of Nigeria because it was not a national study. In Kenya, the Principal Investigator’s institution is APHRC, while local ethical approval and research permits were received from the AMREF Health Africa Ethical and Scientific Review Committee (AMREF-ESRC P564/2018) and the National Commission for Science, Technology, and Innovation (NACOSTI/P/19/16027/27889). All participants were adequately informed about the purpose of the study and methods to be used; anticipated indirect benefits, the lack of direct benefits such as material compensation; potential risks; the right to abstain from participating in the study, or to withdraw from it at any time, without reprisal. Both informed parental consent and adolescent assent forms were obtained from each participant before interviewing the adolescents. Each study participant was given a parental consent form, which was completed by their parents and returned by the adolescents.

### Statistical analysis

Both descriptive and inferential analyses were performed. The univariate analysis involved performing frequency counts and percentages. The bivariate analysis involved performing a Chi-square test and a test of difference (independent t-test and one-way ANOVA), while the multivariate analysis involved performing PCA and Binary Logistic Regression. For the multivariate binary logistic regression, the OR was reported, while the goodness of fit of the model was used to determine whether the application of binary logistic regression followed the required assumption and whether the model sufficiently fits the data. Explanatory variables included in the multivariate model were based on the literature. Only the explanatory variables were statistically significant with the outcome variable at the bivariate level. The Hosmer-Lemeshow test, which checks for goodness of fit for the logistic regression model, was used, with a small Chi-square and a large p-value indicating a good fit to the data.^24^ For our study, the Hosmer-Lemeshow statistics for the combined model was (χ^2^=296.91, p=0.635), the Nigeria model (χ^2^=139.24, p=0.25), and the Kenya model (χ^2^=232.55, p=0.78). Hence, our goodness of fit test based on the p-value obtained indicates that the model adequately fits the data.

## Results

### Descriptive statistics

Table 1 presents the sociodemographic characteristics of the study participants. The mean age of the respondents was 12.0, SD=1.2. (Nigeria 12.0, SD=1.3; Kenya 12.0, SD=1.1). Respondents who were 12 years of age accounted for a higher proportion. While the study participants in Nigeria were evenly distributed by sex, a higher proportion of the participants in Kenya were females (58%).

**Table 1:** Sociodemographic and SRH outcome

Variables	Nigeria (1032)		Kenya (N=880)		Combined (N=1912)	
	Freq	%	Freq	%	Freq	%
Age group						
10–12 years	681	66.0	675	77.0	1356	71.0
13–14 years	351	34.0	205	23.0	556	29.0
Sex						
Male	521	50.5	369	42.0	890	47.0
Female	511	49.5	511	58.0	1022	53.0
Religious affiliation						
Christianity	740	71.7	781	89.0	1521	80.0
Islam	283	27.4	63	7.0	346	18.0
Traditional	9	0.9	36	4.0	45	2.0
Adolescent knows where to get condom						
Yes	114	11.0	174	20.0	288	15.0
No	918	89.0	706	80.0	1624	85.0
Adolescent knows where to get pregnancy prevention methods						
Yes	219	21.2	291	33.0	510	26.7
No	813	78.8	589	67.0	1402	73.3

Freq, frequency; SRH, sexual and reproductive health.

### Gender norm perception

Table 2 presents gender norm perception among the VYA from Kenya and Nigeria. The result of the Chi-Square test revealed significant differences in gender norm perception between the VYA from Kenya and Nigeria (p<0.05) for all the dimensions and sub-domains of gender norms examined. Regional variation suggests a stronger endorsement of these gender norms among VYA in Nigeria relative to their Kenyan counterparts. For instance, for the SDS scale, more VYA from Nigeria scored high relative to their Kenyan counterpart. Similarly, more VYA from Nigeria scored high on stereotypical views about toughness vs weakness, normative heterosexual relations and NRR relative to their Kenyan counterpart.

**Table 2:** Gender norm perception and attitude towards abortion

Gender norm scales	Nigeria (1032)		Kenya (N=880)		Combined (N=1912)	
	Freq	%	Freq	%	Freq	%
Sexual double standard (SDS) scale						
Low	225	21.8	425	48.3	650	34.0
Moderate	323	31.2	325	37.0	648	33.9
High	484	47.0	130	14.7	614	32.1
	χ^2^=225.17, p=0.001					
Stereotypical view about toughness vs weakness scale						
Low	150	14.6	506	57.5	656	34.3
Moderate	472	45.7	266	30.2	738	38.6
High	410	39.7	108	12.3	518	27.1
	χ^2^=417.32, p=0.001					
Normative heterosexual relation (NHR) Scales						
Low	274	26.6	388	44.1	662	34.6
Moderate	344	33.3	284	32.3	628	32.9
High	414	40.1	208	23.6	622	32.5
	χ^2^=82.02, p=0.001					
Normative romantic relation (NRR) subscale						
Low	328	31.8	388	44.0	716	37.4
Moderate	270	26.1	320	36.4	590	30.9
High	434	42.1	172	19.6	606	31.7
	χ^2^=111.16, p=0.001					
Attitude towards abortion						
Adolescent girls who get pregnant should have an abortion if they are not married						
Agree	434	42.0	349	40.0	783	41.0
Neutral	30	3.0	87	10.0	117	6.0
Disagree	568	55.0	444	50.0	1012	53.0
	χ^2^=40.36, p=0.001					
Adolescent girls who get pregnant should have an abortion because they are too young to raise children						
Agree	500	48.4	396	45.0	896	46.9
Neutral	30	3.0	85	10.0	115	6.0
Disagree	502	48.6	399	45.0	901	47.1
	χ^2^=38.31, p=0.001					
Adolescent girls who get pregnant should have an abortion to stay in school						
Agree	510	49.4	430	48.9	940	49.2
Neutral	36	3.5	91	10.3	127	6.6
Disagree	486	47.1	359	40.8	845	44.2
	χ^2^=37.87, p=0.001					

Freq, frequency.

### Attitude towards abortion

The attitude of the VYA towards abortion was statistically significantly different for all the measures between Kenya and Nigeria (table 3). For instance, 42% and 40% of VYA from Nigeria and Kenya, respectively, agreed that adolescent girls who get pregnant should have an abortion if they are not married. Similarly, a slightly higher proportion of VYA from Nigeria than in Nairobi were in support of adolescent girls who get pregnant should have an abortion because they are too young to raise children. However, there was a statistically significant difference in the proportion of VYA from Nigeria and Kenya who agreed that adolescent girls who get pregnant should have an abortion to stay in school.

**Table 3:** Gender norms and abortion perception among VYA

Gender norms	Nigeria			Kenya		
	Attitude towards abortion					
	Disapproval	Neutral	Approval	Disapproval	Neutral	Approval
Sexual double standard (SDS) scale						
Low	33.0	20.0	10.0	54.0	42.0	52.0
Moderate	27.0	34.0	34.0	32.0	42.0	34.0
High	40.0	46.0	56.0	14.0	16.0	14.0
	χ^2^=57.66, p=0.001			χ^2^=11.16, p=0.025		
Stereotypical view about toughness vs weakness scale						
Low	17.0	16.0	10.0	56.0	56.0	62.0
Moderate	43.0	49.0	46.0	32.0	31.0	28.0
High	40.0	35.0	44.0	12.0	13.0	10.0
Normative heterosexual relation scales	χ^2^=11.21, p=0.024			χ^2^=2.73, p=0.605		
Low	39.0	23.0	16.0	49.0	38.0	48.0
Moderate	34.0	35.0	31.0	29.0	36.0	30.0
High	27.0	42.0	53.0	22.0	26.0	22.0
	χ^2^=70.00, p=0.001			χ^2^=10.68, p=0.030		
Subdomains						
Normative romantic relation						
Low	41.5	28.2	24.1	55.0	39.0	40.0
Moderate	25.4	29.0	24.6	31.0	39.0	38.0
High	33.1	42.8	51.3	14.0	22.0	22.0
	χ^2^=34.69, p=0.001			χ^2^=16.98, p=0.002		

VYA, very young adolescent.

### Gender norm perception and attitude towards abortion practice

Table 3 assesses the association between gender norm perception and attitude towards abortion among the VYA. The result revealed a statistically significant association (p<0.05) between all the dimensions and sub-domains of gender norms and abortion perception. More VYA who scored high on the SDS scale, stereotypical views about toughness vs weakness, NRR, and MSP from Nigeria were more likely to be in support of abortion relative to their counterpart from Kenya.

### Multivariate analysis

The result of the multivariate analysis is presented in table 4. The combined model revealed that knowledge of where to get condoms and NRR were the only explanatory variables that significantly predicted attitudes toward abortion. The VYA who knew where to get a condom demonstrated higher odds (OR=1.36, 95% C.I. 0.99 to 1.85) of endorsing abortion among adolescent girls. Also, VYA who scored between moderate (OR=1.49, 95% C.I. 1.06 to 2.06) and high (OR=1.97, 95% C.I. 1.26 to 3.01) on NRR were more likely to endorse abortion relative to those who scored low.

**Table 4:** Binary logistic regression showing predictors of attitude towards abortion

Explanatory variables	Nigeria		Kenya		Combined	
	OR	95% CI	OR	95% CI	OR	95% CI
Sex						
Male	1.000		1.000		1.000	
Female	1.08	0.66 to 1.76	0.88	0.64 to 1.20	0.92	0.71 to 1.19
Adolescent knows where to get condom						
No	1.000				1.000	
Yes	1.07	0.62 to 1.85	1.53*	1.04 to 2.25	1.36*	0.99 to 1.85
Adolescents know where to get pregnancy prevention methods						
No	1.000		1.000		1.000	
Yes	1.26	0.72 to 1.18	1.16	0.83 to 1.63	1.17	0.89 to 1.55
Sexual double standard (SDS) scale						
Low	1.000		1.000		1.000	
Moderate	3.42**	1.25 to 9.31	0.92	0.62 to 1.37	1.14	0.81 to 1.61
High	1.86	0.60 to 5.81	0.57	0.30 to 1.04	0.75	0.46 to 1.22
Stereotypical view about toughness vs weakness scale						
Low	1.000		1.000		1.000	
Moderate	0.78	0.33 to 1.82	1.11	0.77 to 1.59	0.94	0.69 to 1.28
High	1.46	0.61 to 3.49	1.07	0.64 to 1.77	1.28	0.88 to 1.87
Normative heterosexual relation scales						
Low	1.000		1.000			
Moderate	1.49	0.67 to 3.08	0.62*	0.43 to 0.90	0.74	0.53 to 1.02
High	2.58*	1.14 to 5.86	0.84	0.54 to 1.31	1.10	0.76 to 1.60
Normative romantic relationship						
Low	1.000		1.000		1.000	
Moderate	0.94	0.42 to 2.08	1.66**	1.13 to 2.46	1.49*	1.06 to 2.06
High	1.03	0.45 to 2.37	2.48**	1.41 to 4.38	1.97**	1.26 to 3.01

*Statistically significant at 5%.

**Statistically significant at 1%.

Furthermore, factors predicting attitudes towards abortion were found to differ according to country. Among VYA from Nigeria, SDS and NHR were the two gender norm behaviours that significantly predicted attitudes towards abortion. The VYA from Nigeria with moderate scores on SDS were about four times (OR=3.42, 95% C.I. 1.25 to 9.31) more likely to endorse abortion among adolescent girls relative to those who scored low. Similarly, VYA who scored high on NHR were about three times (OR=2.58, 95% C.I. 1.14 to 5.86) more likely to endorse abortion practice when compared with those who scored low.

Among the VYA from Kenya, knowledge of where to get condoms, NHR and NRR significantly predicted attitudes towards abortion. VYA who knew where to get condoms were 1.53 times (OR=1.53, 95% C.I. 1.04 to 2.25) more likely to endorse abortion among adolescent girls. On the other hand, while moderate scores on NHR were associated with lower odds of endorsing abortion (OR=0.62, 95% C.I. 0.43 to 0.90) relative to those who scored low, a high score on NRR was associated with higher odds of endorsing abortion. VYA who scored between moderate (OR=1.66, 95% C.I. 1.13 to 2.46) to high scores (OR=2.48, 95% C.I. 1.41 to 4.38) on NRR were more likely to endorse abortion relative to those who scored low.

## Discussion

Our study found significant differences in gender norm perceptions and attitudes towards abortion for both countries, and these gender norms were significantly associated with attitudes towards abortion. Adolescents, like adults, are influenced by cultural and societal expectations around gender roles and behaviours.^25^ These norms can shape their beliefs and attitudes on various issues,^26^ including abortion. Traditional gender norms may lead to different attitudes towards abortion among adolescent boys and girls. Adolescent boys raised in cultures that emphasise traditional masculinity may be socialised to adopt more conservative views on issues related to sexuality and reproduction, including abortion. They may be encouraged to prioritise notions of responsibility and control, which can lead to more restrictive attitudes toward abortion.^26^ On the other hand, adolescent girls may face expectations related to motherhood and family planning. These expectations could influence their attitudes towards abortion, with some girls feeling pressured to conform to traditional roles and views on reproductive choices. For example, Mmari *et al*^27^ observed that despite the decriminalisation of abortion in Mozambique, young women’s lack of autonomy to decide on pregnancy termination was still a significant barrier to accessing safe abortion services.

Attitudes towards abortion are complex and can vary widely among adolescents. Not all adolescents conform to traditional gender norms, and individual experiences, education, exposure to different life perspectives, and personal beliefs also play significant roles in shaping their attitudes toward abortion. For instance, in a study on stigma and social norms in women’s abortion experiences among young women in Kenya and India, young women demonstrated divergent views concerning abortion. While a few of the participants held positive views, for example, stating that a woman should be able to decide what happens to her body, the majority of the participants expressed their gendered opinion towards abortion, considering women who seek abortion as irresponsible or immoral. Opinions such as the latter are often born from religious views that prohibit premarital sex and/or abortion or from a gendered lens promoting SDS that normalise premarital sex for males as against females.^28 29^

Our study found less acceptance of abortion practice among adolescents in both Nigeria and Kenya, with no significant gender difference, contrary to previous studies, although conducted among the adult population.^16 30 31^ Also, previous studies^9 32^ in both countries have reported less support for abortion. Nigeria and Kenya both have a patriarchal tradition that favours male dominance. Women and adolescent girls constitute the most susceptible demography, facing heightened vulnerability, particularly in terms of poverty within households and communities.^29^ This vulnerability is further compounded by the detrimental cultural perceptions and beliefs regarding gender roles, norms and the empowerment of females. This has continued to fuel gender-based violence, lack of autonomy for women, as well as poor reproductive health indices, including low contraceptive use.^29^ It is not uncommon for adolescents growing up in this environment to be socialised into such norms.^29^ Our study found a stronger endorsement of gender norms among VYAs in Nigeria than in Kenya. While studies have found that children learn gender roles and norms from a very young age,^29 32^ several other factors, including media, can influence the adoption of these norms.

Our study revealed that endorsement of gender roles was significant, with a positive attitude towards abortion. It is also important to note that the influence of gender roles/norms on abortion attitudes has been age-long, with varied results. While there is a dearth of data on research of this nature in Africa, similar studies^33 34^ from the Western world reveal contrary findings where an inverse relationship between the approval of traditional gender roles and support for abortion was observed. It is important to note, however, that none of these studies were conducted among VYA. Also, the gender roles considered in their studies were related more to women’s participation in economic/political activities. Conversely,^35^ found gender norms to be a strong predictor of positive attitude towards abortion, while,^36^ on the other hand, observed that gender norms and attitudes towards abortion are only loosely intertwined. No doubt, there exists a relationship between gender norms and abortion attitudes. However, as posited by Mueller^31^, a more intricate analysis of the specific gender-role attitudes influencing perceptions of women’s roles and rights within society is necessary to better comprehend how these attitudes relate to the opposition or endorsement of abortion.

Research has suggested that adolescents' attitudes towards abortion are influenced by a combination of factors, including gender norms, age, marital status, educational attainment, religion, cultural context, access to information and healthcare services as well as reasons for procuring abortion - people are more likely to have a positive attitude to traumatic abortion rather than elective abortion.^1437^,^40^ As with any societal issue, it is important to consider the diversity of perspectives and experiences among adolescents when examining attitudes toward abortion.

A limitation of this study is the inability to control for an important variable like religion, which often exerts a strong influence on attitude towards abortion and gender norm perception in a conservative countries like Nigeria and Kenya. Notwithstanding, the outcome of this study suggests that the attitude towards abortion might not differ in a conservative context, regardless of gender, and particularly among VYA, although limited evidence exists.

## Conclusion

VYAs are at a critical developmental stage where gender norms and attitudes are learnt and internalised. Gender norms and attitudes play an important role in decision-making processes towards access to SRH services, including abortion services. At an age when behaviours and attitudes are being learnt, it is important for interventions addressing gender and SRH components to inculcate positive attitudes and perceptions about SRH, including abortion. Such interventions should begin at an early age before gender norms and beliefs are turned into practice or behaviour.

## Data Availability

Data are available upon reasonable request.
